# Mechanism Underlying the Weight Loss and Complications of Roux-en-Y Gastric Bypass. Review

**DOI:** 10.1007/s11695-015-1945-7

**Published:** 2015-11-03

**Authors:** G Abdeen, CW le Roux

**Affiliations:** Investigative Science, Imperial College London, London, UK; Diabetes Complications Research Centre, Conway Institute, University College Dublin, Dublin, Ireland; Gastrosurgical Laboratory, University of Gothenburg, Gothenburg, Sweden

**Keywords:** RYGB, Mechanism, Weight loss, Complications, Food preferences

## Abstract

Various bariatric surgical procedures are effective at improving health in patients with obesity associated co-morbidities, but the aim of this review is to specifically describe the mechanisms through which Roux-en-Y gastric bypass (RYGB) surgery enables weight loss for obese patients using observations from both human and animal studies. Perhaps most but not all clinicians would agree that the beneficial effects outweigh the harm of RYGB; however, the mechanisms for both the beneficial and deleterious (for example postprandial hypoglycaemia, vitamin deficiency and bone loss) effects are ill understood. The exaggerated release of the satiety gut hormones, such as GLP-1 and PYY, with their central and peripheral effects on food intake has given new insight into the physiological changes that happen after surgery. The initial enthusiasm after the discovery of the role of the gut hormones following RYGB may need to be tempered as the magnitude of the effects of these hormonal responses on weight loss may have been overestimated. The physiological changes after RYGB are unlikely to be due to a single hormone, or single mechanism, but most likely involve complex gut-brain signalling. Understanding the mechanisms involved with the beneficial and deleterious effects of RYGB will speed up the development of effective, cheaper and safer surgical and non-surgical treatments for obesity.

## Introduction

The Roux-en-Y gastric bypass (RYGB) includes a small gastric pouch (15–30 mL) on the lesser gastric curvature [1, 2] which is completely divided from the gastric remnant and then anastomosed to the jejunum (leaving an alimentary or Roux limb of typically 100–150 cm). The size of the gastro-jejunal anastomosis is controversial as initially it was thought that an element of restriction may be helpful in slowing the progress of food from the oesophagus into the jejunum, but more recently the aim has been rapid transit of food into the jejunum to generate the gut signals to reduce meal size [3]. Bowel continuity is restored by an entero-entero anastomosis between the excluded biliopancreatic limb (BPL) and the alimentary limb. This anastomosis is usually performed 100–150 cm distal to the gastro-jejunostomy, although it has also been performed up to 250 cm distally in an attempt to create calorie malabsorption [1]. Usually, the BPL is approximately 50 cm, but since other operations such as the biliopancreatic diversion or mini-gastric bypass with much longer BPL have greater reduction in insulin resistance, renewed interest in the length of the biliopancreatic limb BPL has developed [4].

Operative times vary between 45 and 90 min and the average hospital stay is 1–3 days, although same-day discharge following RYGB procedure have been successful [5]. Early complications, within 30 days after surgery, do occur in approximately 4 % of patients and include bleeding, perforation or leakage, which need immediate surgical re-intervention [6]. Late complications such as significant abdominal pain, small bowel obstruction, anastomotic stenosis or marginal ulceration can occur in 15–20 % of patients after 30 days from surgery to over 10 years, and surgery or endoscopic therapy is often used for both diagnosis and/or treatment [7].

Even though RYGB does not treat some of the aetiological factors of morbid obesity, such as the obesogenic environment we live in, it does successfully result in 20–30 % long term, over 2 years of weight loss and maintenance [8–10], in addition to an improvement or remission of many obesity-related co-morbidities [11–15] such as hypertension, type 2 diabetes mellitus, obstructive sleep apnoea and musculoskeletal pain. Approximately 40 % of obese patients with type 2 diabetes go into remission within days or weeks after RYGB [16].

The RYGB is the best studied procedure regarding underlying mechanisms. The aim of this review is to describe the mechanisms through which RYGB surgery enables weight loss for obese patients and helps in understanding its complications by using observations from both human and animal studies.

## Food Intake

### Research Studies

#### Hunger and Fullness

Lifestyle changes with a lower calorie diet can be effective at initiating weight loss; however, most of the results from randomised controlled trials (RCT) are disappointing regarding long-term weight loss maintenance [17, 18]. Approximately 70–80 % of patients fail to maintain their initial lifestyle-induced weight loss thought to be due to physiologically compensatory responses that defend the previous weight ‘set point’ [19]. Whilst on a long-term low-calorie diet, patients usually report an increase in hunger, a decrease in satiety and pre-occupation with energy-dense fatty and sweet food [20, 21]. This may be part of a normal physiological response and not due to lack of motivation.

Reduced calorie intake after RYGB is usually a consequence of significantly smaller meal sizes, and reduced calorie content of food eaten [22] compensated only partially by increased meal frequency [23]. Enhanced satiety is the dominant contributing factor [24]. A dramatic decrease in daily energy intake, 600–700 kcal [22, 25], during the first month post-surgery increases to 1000–1800 kcal during the first year [22, 26–32]. An average reduction of 1800 kcal per day from pre-operative intake can be sustained for several years [32, 33]. Protein intake during the first year after surgery is often lower than recommended at 0.5 g/kg, rather than the recommendation of at least 1.5 g/kg/day [27, 34]. The mechanisms are unclear, but may be due to temporary intolerance of higher protein diet and dairy foods [22, 25, 27, 35–37]. Relative intake of fat and carbohydrates decrease during the first year post-surgery, but return to the baseline after 1 year [22], although the contribution of high and low glycaemic index carbohydrates may change. Many patients reduce their intake of high glycaemic index carbohydrates and increase their intake of lower glycaemic index carbohydrates. Changes in behaviour associated with eating after RYGB were reported in the 1970s using structured interviews that suggested that patients reached satiety more quickly, with the most common reason given as a ‘lack of desire’ for food [38].

### Potential Mediators

#### Increased Transit of Food into the Midgut Through the Gastric Pouch

Whether the size of the gastric pouch and stoma in RYGB surgery affects food intake and body weight is contested. It remains controversial in both the human and animal literature whether a larger gastric pouch and stoma causes less weight loss [39–43]. The stoma becomes more ‘compliant’ with time, allowing food to transit more easily from the pouch into the alimentary limb, but may also result in food being ‘stored’ in the pouch and not emptying rapidly enough. Thus, the initial diameter of the anastomosis may not affect weight loss in the long term [44]. To study a RYGB technique that created a very small pouch, a high-pressure manometer was used, but a large stoma demonstrated that the pressure in the pouch (immediately proximal to the gastroenteral anastomosis) was *lower* than in the alimentary limb [45]. This suggests there was no restriction at the level of the stoma because of the absence of a high-pressure zone proximal to the pouch. Insertion of a gastric balloon into the alimentary limb and inflation of the balloon to a pressure of 20 cm water demonstrated that patients with the highest pressure generated by the alimentary limb had the smallest meal volume during an ad libitum meal. In contrast, those with the lowest pressure in the alimentary limb took longer to terminate their meal. Mechanoreceptors within the alimentary limb may be important determinants of meal size if food rapidly transits through the pouch to reach the alimentary limb in a less digested state than usual. The component that determines caloric intake may be the alimentary limb and not the pouch size or stoma diameter.

#### Hormonal

RYGB alters endogenous gut hormone responses to a meal. Glucagon-like peptide-1 (GLP-1), peptide YY (PYY) and ghrelin have been the best studied candidates in the context of reduced food intake and sustained weight loss after RYGB. GLP-1 and PYY responses to mixed meals or oral glucose have been at the centre of interest of several studies investigating patients 6 weeks to 10 years after RYGB [46–51]. Significantly elevated responses are seen in GLP-1 and PYY as early as 2 days after RYGB [52] and may remain elevated for more than a decade after RYGB [53]. Patients who lost the most weight after RYGB also had the highest levels of these postprandial satiety gut hormones [54, 55]. Blocking the release of these hormones in humans and rats with octreotide increased food intake after RYGB, but not after adjustable gastric banding (AGB) surgery in humans [51] or sham operations in rats [56].

Mechanistic studies in rodents have suggested the physiological significance of PYY because weight loss in PYY-knockout mice after a RYGB variant was lower than in wild-type mice [57]. Exogenous PYY specific antibodies also increased food intake in rats after bypass type procedures [51]. Physiologically, PYY has been shown to delay gut transit time, but probably does not increase energy expenditure in human [58]. GLP-1 responses are very similar to those of PYY after RYGB, but have additionally been linked with increases in insulin secretion [59, 60]. Postprandial responses of GLP-1 before surgery do not correlate with change in weight loss after surgery, suggesting that pre-operative gut hormone responses are not prognostic [61]. Enhanced GLP-1 signalling on its own is also not sufficient to reduce body weight after RYGB, suggesting that it is multiple gut hormone responses that mediate the increased satiation after a meal [62].

Reduced ghrelin was the first proposed hormonal mechanism to explain weight loss after RYGB. At first, ghrelin levels were thought to be lower compared to diet-induced weight loss which increased ghrelin in a control group of subjects [63]. It was postulated that this decrease was partially responsible for reduced hunger after RYGB. Subsequent studies in patients after RYGB were more controversial reporting a reduction in fasting and postprandial ghrelin levels [50, 64–70], no alteration in fasting and postprandial levels [51, 52, 71–79] and a rise in fasting ghrelin levels [80–84]. Considering all the data and variability, it is likely that RYGB results in a comparative ghrelin deficiency considering that ghrelin normally increases after diet-induced weight loss, but the magnitude of this contribution is unclear [85, 86].

#### Neural

The vagal afferent fibres in the gastric and proximal small bowel mucosa are known to be sensitive to mechanical stretch in order to detect the volume of ingested food [87]. The vagus nerve with both the ventral and dorsal gastric branches on the large gastric remnant is transected during the formation of the gastric pouch. The vagal fibres to the gastric pouch are thus intact, and these could mediate satiety as food passes through the pouch. The vagal denervation more distally may attenuate signalling. Taken together, this may play a role in satiation [88]. Visceral sensory information from the gut is communicated centrally using the afferent (sensory) vagus nerve signalling to the nucleus of the tractus solitarius (NTS). Here, visceral sensory information and hormonal and metabolic inputs are integrated together with neuronal inputs from other brainstem areas [89] and may well be the most important way in which RYGB signals to the brain. Transmission of these signals involving the gut hormones such as ghrelin may be impaired after vagotomy [90]. RYGB appears to have the potential to alter neural responses [91] to reduce hedonic behaviour associated with eating highly palatable and calorie-dense foods. These changes in reward value of food may alter the amount of food consumed [38, 92–94].

#### Change in Bile Acids

Bile acids are agonists for the cell-membrane G protein-coupled receptors, TGR5, which in turn enhances the release of GLP-1 and PYY. Bile acids also bind the farnesoid X receptor (FXR) [95]. The anatomical changes after RYGB result in bile progressing down the biliopancreatic limb to the distal L cells without mixing with food. As a result, the availability of undiluted bile acids in the distal intestine may enhance stimulation of TGR5 receptors on L cells [96]. Serum bile acid concentration is raised after RYGB [97] and is associated with increased energy expenditure possibly through signalling via the cyclic adenosine monophosphate cAMP-dependent thyroid hormone triggering enzyme type 2 iodothyronine deiodinase [98]. Fibroblast growth factor (FGF) 19 is increased and binds to fibroblast growth factor receptor (FGFR4) activating fibroblast growth factor receptor c-kit (FGRR1c) in the presence of co-receptor β Klotho [99]. The result is increased protein synthesis in the liver [100]. FGF19 also plays a role in enhanced mitochondria activity [100]. Activation of the FXR receptor may facilitate the effects of bile acids on energy homeostasis through FGF19 that is released from ileal enterocytes which can lead to increases in metabolic rate and decreases in adiposity [101, 102].

Bile acids, after a mixed test meal in human subjects, was positively correlated with circulating GLP-1 and PYY, but negatively correlated with ghrelin [103]. Pournaras et al. have demonstrated that total plasma bile acids are elevated after RYGB [104] and suggested that they may be partly responsible for the intestinal hypertrophy, anorexigenic hormone secretion and alterations in gut microbiota [105].

#### Change in Gut Microbiota

Obesity is associated with low-grade inflammation, increased Firmicutes and decreased Bacteroidetes in animals [106] and humans [107–109]. Intestinal microbiota has also been shown to utilise energy from food and thus increase the host’s energy-harvesting capacity [110]. Proteobacteria (gammaproteobacteria) has been shown to increase after RYGB in humans [111] with the major contributor being *Enterobacter hormaechei*. The significant improvement of weight, inflammation and metabolic status after surgery was associated with increased bacterial variety. An association was observed between adipose tissue gene expression and bacterial genes at baseline with a 10-fold increase 3 months after surgery, and this may suggest a restored crosstalk between both the gut microbiota and the host [112].

After RYGB, acidity was reduced in the alimentary limb leading to a decrease of hydrochloric acid flux in the gut, while bile acids were increased in the biliopancreatic limb. Bacteroidetes growth was attenuated at lower pH, whereas *Escherichia coli* increased at a higher pH. Gut microbiota quickly adapt in a ‘starvation-like state’ created by RYGB and rapidly and sustainably increase. Changes in microbiota in mice after RYGB were independent of weight alteration and caloric restriction [113]. Transfer of the gut microbiota from RYGB-treated mice to non-operated, germ-free mice resulted in weight loss and reduced fat mass in the recipient animals. The altered microbial production of short-chain fatty acids that increases may partly be an explanation [113]. Although RYGB did not change gut microbiota from the ‘obese state’ to the ‘lean state’, it did create a ‘third state’ which on balance appear to be associated with many of the beneficial characteristics of RYGB.

## Food Preferences

### Observations

Weight gain has been linked to a preference for both sweet and/or high-fat foods [114, 115], which may partly explain why obese people regain body weight frequently after ‘dieting’ [116, 117]. The common view summarised earlier by Pangborn and Simone is: “In the mind of a normal person, sugar and sweets are ‘fattening’ and most overweight people have a ‘sweet tooth’” [118]. Hedonism associated with palatable foods is considered a significant factor which increases the prevalence of obesity. A motivational factor that is referred to as ‘hedonic hunger’ [119] may be a trigger for overeating [120].

Patients after RYGB tend to increase the intake of fruit and vegetables as well as low-fat food [121, 122]. The dumping syndrome was thought to induce these changes in food preference [123], as initially it was considered as a useful characteristic of the RYGB to ‘teach’ patients to avoid calorie-dense foods and thus consume fewer calories [124]. However, patients after RYGB appear to make healthier food choices and adopt a more balanced diet (even when they do not experience dumping) [121, 125] and have considerable reduction in energy intake (EI) and energy density. A comparison on food groups was done for a group of patients after RYGB and total number of servings from fat, grains and sweetened beverages was reduced and remained reduced in the longer term. However, meats, dairy products, fruits and sweets were reduced in the short term, but then returned to baseline by 12 months [22]. When energy intake was reduced to 1300 kcal, 60 and 25 % of patients ,respectively, were consuming less than one serving per day from both fruits and vegetables. Whole grains intake increased from 25 to 40 % within the first 3 months, but then returned to baseline at 12 months [22]. The association between reduced diet energy density and weight loss is controversial as some studies describe no association [126], while others show that shifts in food preferences are partially responsible for the decreased calorie intake and weight loss after RYGB [127].

RYGB in humans appears to alter taste through unconditional and conditional mechanisms [24, 128–130] leading to the concept of ‘behaviour surgery’ [123]. In 1987, Sugerman et al. reported that ‘sweet-eaters’ did particularly well after RYGB [131, 132]. Some of the initial findings were confounded by intolerance to sweets related to symptoms of the dumping syndrome [38, 131–133]. Conditioned taste aversion may thus be a factor in some patients. These initial assumptions resulted in many clinicians thinking that the RYGB works by ‘punishing’ the ‘poor behaviours’ of obese patients. The notion that RYGB becomes an external enforcer that goes against the free will of the patient has led to some authors questioning the morality of RYGB as a tool that changes patients’ behaviour against patients’ natural wishes [134]. This misconception may have reduced the wider acceptance of RYGB as a valid physiological treatment for the pathology that results in obesity. Classical *conditioned food aversion* is, however, an unlikely explanation as most patients with severe dumping still report that they like the taste of sweet foods, but that they have learned to consume only small quantities that do not cause negative visceral symptoms or consume sweets at night before bedtime, suggesting a *conditioned food avoidance* to be a more likely explanation. Distinguishing between the terms is important because avoidance implies that the palatability of sweet or fat did not change when small quantities are consumed, but that the subject ‘learns’ to stop consuming the food sooner (earlier avoidance) because large quantities may have negative visceral consequences [135–137].

### Mediators

RYGB could be exerting its effects on food selection and preference through any one of the taste function domains important in normal physiology such as sensory-discriminative (stimulus identification), hedonic (ingestive motivation) and physiological (digestive preparation) [138, 139]. Affective responses to taste stimuli, which can be considered an example of ingestive motivation, can be both conditioned and unconditioned. It remains controversial which of these three domains are involved and what their interactions are to determine food preferences after RYGB surgery. For example, RYGB could have effects directly on the central gustatory pathways related with feeding and reward through gut hormonal mediators. Alternatively, changes in the sensory signals could alter the intensity or the quality of tastants, but also lead to an unconditioned change in palatability. If RYGB causes visceral malaise after ingestion of fat, then it is possible that the palatability of fat could alter through a process of learning (conditioned response) [140].

Although there are suggestions in animal models that the hedonic properties of sweet and fat stimuli may change after RYGB [23, 140–144], less work has been done in humans. Miras et al. using the progressive ratio task showed that RYGB resulted in the selective decrease of the reward value of a sweet and fat tastant, but not vegetables [145]. Further support comes from studies of brain reward cognitive systems linked to eating behaviour as studied by functional MRI (fMRI), where brain hedonic responses to calorie-dense food are lower after RYGB compared to patients who have lost similar amounts of weight after adjustable gastric banding [128].

## Energy Expenditure

According to the laws of thermodynamics, energy that enters a system (energy intake) must either be stored (body energy gain) or be used (activity, heat or faecal energy loss). Energy expenditure (EE) is usually decreased during food restriction, a phenomenon known as the ‘starvation response’ [146]. Weight loss in rodent models of RYGB is associated with preservation of lean body mass and increased EE [146]. Humans have decreased basal metabolic rate, but increased meal-induced thermogenesis after RYGB [32, 122, 147–153]. Evidence is now also emerging to suggest that the metabolic rate of the small bowel is increased after RYGB with more carbohydrate consumption which may explain the changes observed in respiratory quotient after these operations [154]. Reduced resting energy expenditure (REE) or basal metabolic rate after RYGB [122, 147, 155–157] may be attenuated due to relative lean mass preservation. Patients who regain the weight they lost 2 years after RYGB have lower REE [149], suggesting that elevating REE after RYGB may enhance weight loss. Physical activity may further help increase activity-related EE and also preserve lean mass, and therefore REE, after RYGB [158].

## Calorie Malabsorption

Several bariatric operations were designed to result in malabsorption of calories [159]. The exclusion of the approximately 10 % of the bowel (50 cm of BPL) after RYGB is unlikely to result in calorie malabsorption usually during other small bowel resections. Moreover, the exaggerated gut hormone responses which reduce gut transit have a net result of RYGB not altering oro-caecal transit time or functional enterocyte mass [16]. RYGB may, however, impair pancreatic exocrine function which could contribute to a small amount of fat malabsorption, the magnitude of which is probably too small to contribute substantially to weight loss [160–162].

## Mechanisms of Complications

The rise in the number of RYGB procedures [163] has also increased the absolute number of complications associated with this procedure even though the percentage of patients with complications has reduced due to better surgical experience [164].

Postprandial hypoglycaemia, even in patients who never had type 2 diabetes, can occur several hours after a meal and is distinct from early dumping syndrome which occurs within minutes after eating [165, 166]. Early dumping is an outcome of rapid emptying of food into the jejunum due to the lack of a pylorus presumably causing neural activation in the proximal alimentary limb [167]. Late dumping, or ‘postprandial hypoglycaemia’, happens 1–3 h after ingesting a meal and is a result of the exaggerated insulin response to high glycaemic index carbohydrates in the meal. The proposed mechanisms involve increased β-cell mass and improved β-cell function and non-β-cell mechanisms, which may include a lack of ghrelin (a counter-regulatory measure to hypoglycaemia) [63, 168]. In addition, the sustained weight loss can reduce insulin resistance which renders the previous insulin responses needed pre-surgery to suddenly become excessive. The aetiology of hypoglycaemia is likely to be different for individual patients and is also probably a mixture of the anatomic, hormonal and metabolic changes after RYGB [169]. Although treatment of this complication can be difficult, pancreatectomies are no longer advised [170], but rather a multimodal medical approach is favoured [171].

### Unexplained Abdominal Pain

Up to 10 % of the patients complain of unexplained chronic abdominal pain which can be difficult for both the treating clinician and patient to acknowledge [172, 173]. Mild abdominal pain is reported by up to 95 % of patients at some point after RYGB [172, 174–176]. Symptom severity fluctuates between vague discomfort and severe colicky pain [177]. Vomiting and nausea, especially if prolonged, are symptoms of pathology and are not part of the normal postoperative course after RYGB; nonetheless, up to 80 % of patients report the symptoms at some point after surgery [172, 174]. Abdominal pain may be recurrent, and it should be remembered that internal hernias may spontaneously reduce causing intermittent pain. Early investigation when acute symptoms of abdominal pain first presents is mandatory due to the risk of obstruction, volvulus and ischaemia of the herniated bowel [172, 178]. Cross-sectional imaging is often unhelpful and the use of laparoscopy is frequently required for diagnosis. Management protocols for chronic unexplained abdominal pain are not clearly defined, but the jejunal-jejunal anastomosis is currently receiving more attention as a possible cause for these chronic problems.

### Anastomotic Stenosis

With the circular stapler technique, this can be a common complication with a reported incidence of up to 27 % and a recurrence rate of up to 33 % [179]. Usually dysphagia occurs within 6 months after surgery. Endoscopy can often be used both as a diagnostic but also an intervention tool.

### Vitamin Deficiencies: Iron, Vitamin B12, Folic Acid, Vitamin D and Calcium

Iron deficiency occurs in up to 49 % of patients after RYGB [180]. Reduced acid production in the small stomach pouch decreases iron absorption [181]. For iron to be absorbed, the ferric iron in foods has to be reduced to the ferrous state, but because hydrochloric acid is lower after RYGB, this process is attenuated [182]. Reduced intake of iron-rich foods after RYGB such as red meat may also contribute [183, 184].

In the stomach, both pepsin and hydrochloric acid are required for absorption of vitamin B12. Deficiencies of vitamin B12 occur in up to 70 % of patients after RYGB [184–186] because achlorhydria prevents vitamin B12 separation from foods due to reduced ingestion of meat and insufficient secretion of intrinsic factor after surgery [182].

Folic acid deficiency affects up to 35 % of patients after RYGB. Folate absorption is enabled by hydrochloric acid with absorption in the proximal third of the small bowel most important [186]. Vitamin B12 also acts as a coenzyme in converting methyltetrahydrofolate to tetrahydrofolate. Thus, folate deficiency might result from achlorhydria, bypassing of the proximal small bowel, vitamin B12 deficiency and/or decreased folate ingestion [184–187].

Hypocalcaemia occurs in up to 10 % and low serum 25-hydroxy vitamin D levels in up to half of RYGB patients [188]. Nevertheless, most obese patients had significantly lower basal 25-hydroxyl vitamin D concentrations and higher parathyroid hormone concentrations as compared to age-matched lean controls [189]. Deficiencies may occur because calcium is typically absorbed in the proximal small bowel which is bypassed after RYGB. Intolerances can develop to calcium-rich sources such as milk especially if the fat content is high. Calcium can be released from bone as evident from the increased bone turnover and subsequent reduced bone mass after RYGB [190, 191]. The higher bone turnover in the RYGB patients could be partly due to the weight loss in these patients [192], but animal studies suggest that bone loss exceeds what would be expected from weight loss alone [193].

### Loss of Bone Density

Many patients with obesity have very healthy bone density before surgery due to long-term excessive weight bearing. This may be protective and partly explains the controversy of why the loss of bone density after RYGB does not cause more bone fractures [194, 195] even if the risk for fracturing may be increased. Multiple mechanisms may contribute to RYGB reducing bone density, including physiologically reduced mechanical load related to weight loss after surgery, hyperparathyroidism due to insufficient calcium consumption or reduced intestinal calcium and vitamin D absorption. Humoral factors from adipose tissue (oestradiol, leptin, adiponectin), pancreas (e.g. insulin, amylin) or the gut (ghrelin, glucagon-like peptide-2, glucose-dependent insulinotropic peptide) may also play a role [196, 197] by connecting a web of consistent regulatory pathways [196].

### Kidney Stones

Hyperoxaluria is common after RYGB, but the incidence of renal calculi is much lower than after jejunal-ileal bypass (JIB) [198–200]. Comparison with the JIB is important because the incidence as well as the potential mechanisms may be different after RYGB. The lithogenic effects after RYGB may stem from reduced calcium binding to oxalate in the intestinal lumen. The excess oxalate is then cleared by the kidneys resulting in hyperoxaluria and calcium oxalate nephrolithiasis. Almost 21 % of patients after JIB, which causes significant malabsorption, developed kidney stones 5 years after surgery [201], but the incidence of kidney stones after RYGB appears to depend on a combination of other factors such as hydration status and urine volume [200]. Patients in high stone-forming areas of the world have increased number of stones while those in low stone-forming countries may have an incidence similar to the background population [202]. Thus, RYGB alone is not enough to cause kidney stones, but it does potentiate other predisposing factors.

## Conclusion

RYGB confers both benefits and complications, the mechanisms of which are still only partially understood. Most, but not all, clinicians would agree that the beneficial effects outweigh the harm that may be caused [203]. The exaggerated release of the satiety gut hormones with their central and peripheral effects on glycaemia and food intake [52, 75, 204] has given new insight into the physiological changes that take place after surgery. The initial enthusiasm after the discovery of the role of the gut hormones may need to be tempered as the magnitude of the effects of these gut hormones on weight loss may have been overestimated. The physiological changes after RYGB are unlikely to be due to a single hormone, or single mechanism, but are more likely to additionally involve complex gut-brain nutrient and neural signalling [205, 206]. Understanding these mechanisms will speed up the development of more effective and safer surgical and non-surgical treatments for obesity.

All studies that are mentioned in the review adhered to the expected high level ethical considerations and were approved by the appropriate institutional review board.
